# Risk factors for development of lower limb osteoarthritis in physically demanding occupations: A narrative umbrella review

**DOI:** 10.1002/1348-9585.12103

**Published:** 2019-12-11

**Authors:** Ben Schram, Robin Orr, Rodney Pope, Elisa Canetti, Joseph Knapik

**Affiliations:** ^1^ Tactical Research Unit Bond University Robina QLD Australia; ^2^ School of Community Health Charles Sturt University Albury NSW Australia; ^3^ Uniformed Services University Bethesda Maryland

**Keywords:** lower limb, military, occupation, osteoarthritis, work

## Abstract

**Objectives:**

Osteoarthritis (OA) is a common disorder which affects the joints. As relationships between occupational factors and lower limb OA have been widely studied in systematic reviews, the aim of this umbrella review was to synthesize their key findings in the risk factors for development of lower limb OA.

**Methods:**

A systematic search was conducted using the databases PUBMED, Cumulative Index of Nursing and Allied Health Literature, and Elton B Stevens Company to identify reviews examining associations between lower limb OA and occupational tasks. These reviews were rated for their methodological quality before key data were extracted and synthesized.

**Results:**

Sixteen reviews were found, seven pertained to the knee, four to the hip, two to a variety of joints, and three to both the hip and knee. One was deemed to be of *high* methodological quality, one of *critically low* methodological quality, and the others of *moderate* methodological quality. The reviews found moderate to good evidence for heavy occupational lifting to be associated with an increased risk of OA at the knee and the hip. Kneeling, squatting, and climbing, previous injuries to joints, being overweight and obese were also predictive of lower limb OA.

**Conclusion:**

Occupations which involve heavy physical workloads increase the risk of developing lower limb OA. Heavy lifting, squatting, knee bending, kneeling, and climbing may all increase the risk of developing OA in both the knees and hips. Efforts to reduce exposure to these tasks, reducing joint injuries, optimizing bodyweight may reduce the risks of lower limb OA for occupations which are physically demanding.

## 1 BACKGROUND

Osteoarthritis (OA) is one of the most common disorders which affect the joints of the body, and is the most common form of arthritis.^1^ OA is manifested by joint pain, aching, stiffness, functional limitation, and progressive disability.^2^ The diagnosis of OA is based on clinical and radiographic criteria.^3^ Clinical diagnosis of OA is made through both the history and physical examination of the presenting person, typically considering components of the American College of Rheumatology classification of joint pain and ensuring at least three of the following six features are present: age >50 years, morning stiffness lasting greater than 30 minutes, crepitus in the joint, bony tenderness, bony enlargement, and no palpable warmth emanating from the joint.^4^ Radiographically, the World Health Organisation defines OA as a joint presenting with osteophyte formation, joint space narrowing, sclerosis, and cysts.^5^ These radiographic changes are typically graded using a scheme devised by Kellgren and Lawrence,^6^ referred to as the K/L grading system, whereby a score over two (Grade 2) is indicative of OA being present. A Grade 2 is assigned where there is 50%‐75% joint space narrowing without secondary features of osteophytes and subchondral sclerosis,^7^ although research does suggest that the K/L grading system wording is inexact and open to interpretation.^8^ However, and similar to some other pathological conditions, imaging findings do not correlate well with symptoms.^4^ For example, Anderson and Felson 9 found that individuals with what was considered to be “moderate” knee OA on imaging were symptomatic in only 40% of cases, while those considered to have “severe” OA based on imaging results were symptomatic in only 60% of cases.

The prevalence of OA is on the increase and this is thought to be at least in part due to increasing rates of overweight and obesity, associated with increasingly sedentary lifestyles.^3^ OA of the hip and knee in particular constitute one of the greatest contributors to global disability from musculoskeletal diseases,^1^ with an estimated 20% of individuals over 60 years of age having already undergone, or seeking a hip or knee joint replacement due to severe pain from OA.^10^ Reported risk factors for the development of OA in the general population include older age, female gender, being overweight or obese, previous injury, involvement in competitive sports, and high levels of exposure to occupational factors which load or cause trauma to joints.^10^, ^11^, ^12^, ^13^ Given the physically demanding nature of military service, the common military requirement to carry heavy loads,^11^, ^14^ and the higher rates of acute injury observed in military populations when compared to the general population,^15^ it could be expected that military personnel will be at greater risk of developing OA than the general public. In fact, recent reviews 11, 16, 17, 18 have indicated a disproportionately high incidence of OA, which is rising, among military service members when compared to the general population. Despite being one of many physically demanding occupations and quite diverse, military personnel are required to learn and maintain basic skills. Physical requirements of military service have been broadly categorized as lifting and carrying, lifting and lowering, climbing, digging, walking, marching and running, and pushing and pulling.^19^

The knee is the most commonly affected joint in military personnel 11, 20 and issues arising from OA have presented as the most common or second most common (depending on the year) cause of discharge from United States (US) military service for over a decade.^21^ US figures indicate that across all active duty service members, incidence rates for OA are approximately 7.9 cases per 1000 person‐years,^20^ with higher incidence rates in the Army (9.9 per 1000 person‐years) than in the Air Force (7.0 per 1000 person‐years), Navy (4.6 per 1000 person‐years), and Marine Corps (4.0 per 1000 person‐years).^16^ Given the extent to which OA affects military personnel, the follow‐on effects for medical discharge and physical readiness, and the preponderance of lower limb joints affected by OA in military personnel, the aim of the current narrative umbrella review was to identify, critically appraise, and synthesize key findings from previous literature reviews that have examined risk factors for the development of lower limb OA in physically demanding occupations to inform future research, prevention, and management of lower limb OA in the military context.

## 2 METHODS

A systematic search was conducted for published literature reviews in the PubMed, Cumulative Index of Nursing and Allied Health Literature, and Elton B Stevens Company databases (November 2018) using dedicated, but comparable search terms for each database (Table 1). Search results were screened by title to remove reviews that were clearly not relevant. For the reviews remaining, abstracts and full texts were subsequently obtained and subjected to eligibility appraisal using dedicated inclusion criteria. Articles were included if they were: (a) a literature review (either narrative or systematic), (b) published within the preceding 15 years, (c) written in English, (d) reviewed studies involving human participants, (e) subjected to peer‐review, and (f) investigated risk factors for development of lower limb OA in personnel from physically demanding occupations.

**Table 1:** Details of literature search including databases used, search terms, and filters

Database	Search terms	Filters
PubMed	("arthritis"[Title/Abstract] OR "osteoarthritis"[Title/Abstract]) AND ("ankle"[Title/Abstract] OR "knee"[Title/Abstract] OR "hip"[Title/Abstract] OR "foot"[Title/Abstract] OR "lower limb"[Title/Abstract]) AND ("risk"[Title/Abstract] OR "prevalence"[Title/Abstract] OR "cause"[Title/Abstract])	Full text, 2003‐2018, In English, on Humans, Reviews
CINAHL	(AB) Arthritis OR osteoarthritis AND (AB) ankle OR knee OR hip OR foot OR lower Limb AND (AB) risk OR prevalence OR cause	Human, peer reviewed, from 2003, in English, Reviews
EBSCO	Arthritis OR osteoarthritis AND ankle OR knee OR hip OR foot OR lower Limb AND risk OR prevalence OR cause	Human, peer reviewed, from 2003, in English.

Abbreviations: CINAHL, Cumulative Index of Nursing and Allied Health Literature; EBSCO, Elton B Stevens Company.

The methodological quality of the included reviews was critically appraised using A MeaSurement Tool to Assess systematic Reviews (AMSTAR) 2.^22^ The AMSTAR 2 is a 16‐question instrument which is used in assessing the methodological quality of systematic reviews of both randomized and non‐randomized studies. The instrument is not designed to give an overall score, only an overall rating of the level of confidence in the results of a review (ie, critically low, low, moderate, and high). A rating of high is given to a review which has no or one non‐critical flaw and is therefore deemed to be an accurate and comprehensive summary of the results. A rating of moderate is given to a review with more than one weakness, however, with no critical flaws. A rating of low is given to a review with a major critical flaw and therefore may not provide an accurate and comprehensive summary. Finally a critically low review has more than one critical flaw and is deemed to be unreliable to provide an accurate and comprehensive summary of the literature.^22^ To minimize scoring bias, two raters independently (BS and EC) scored each review on the AMSTAR 2. To determine the final score, discrepancies in scoring were discussed and a final score agreed upon by consensus. Where consensus in score differences could not be obtained, a third author (RO) adjudicated to establish a final score. Given that the AMSTAR 2 was designed for systematic reviews, narrative reviews were not rated.

Key findings of the included reviews that were relevant to the aims of this umbrella review were extracted, summarized in tabular form, and synthesized using a structured, narrative synthesis approach. Types of data extracted from the reviews included author and year, type of review, number of studies included, the focus of the review, and the key findings of the review. Findings were weighted in the narrative synthesis based on the methodological quality of each source.

## 3 RESULTS

### 3.1 Search, screening, and selection outcomes

**Figure 1:**
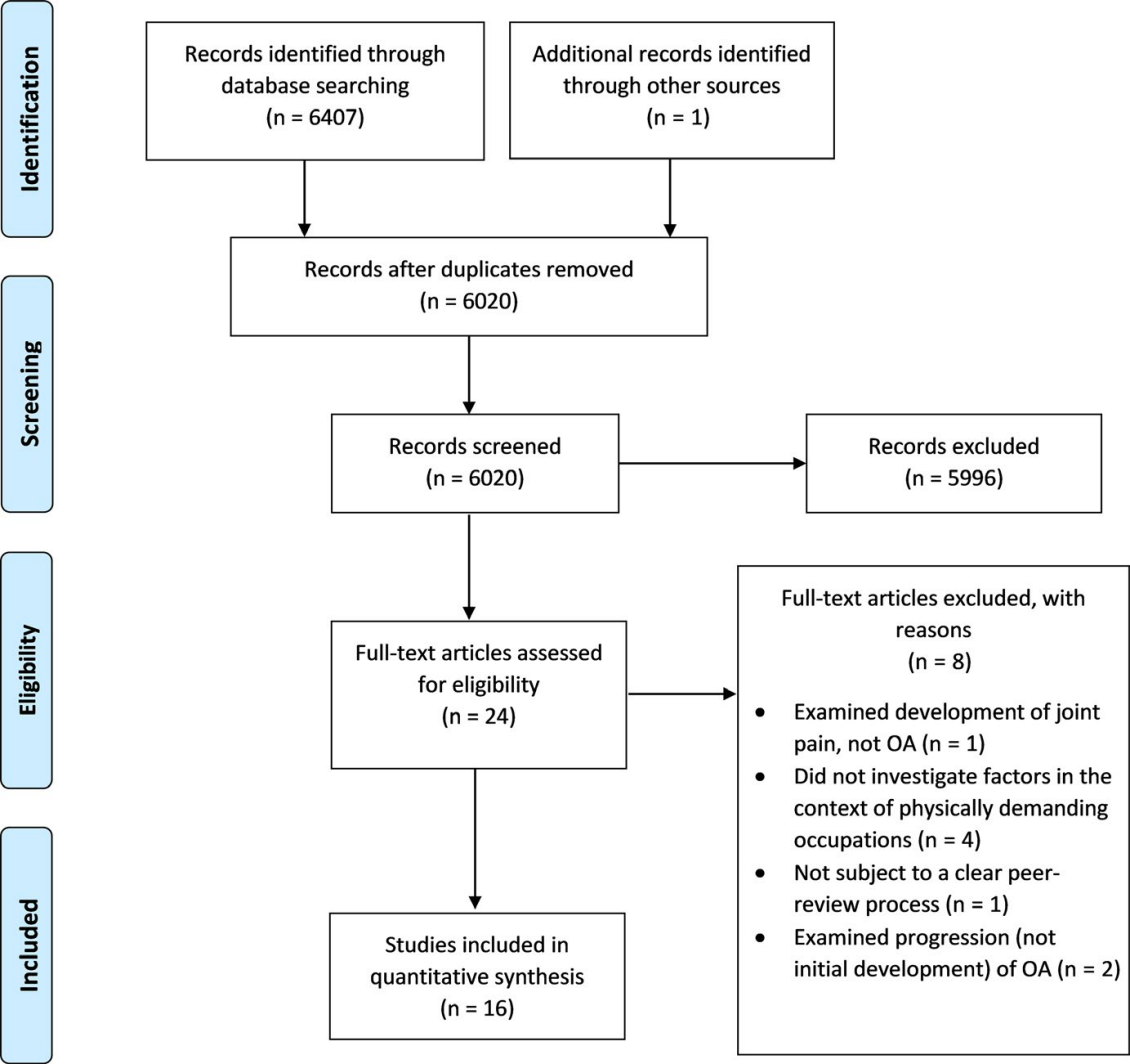
PRISMA diagram showing results of the search, screening, and selection processes

From an initial 6408 identified articles, 388 duplicates were removed along with an additional 6004 which did not meet the eligibility criteria for inclusion (Figure 1). Key findings from the 16 included reviews are summarized in Table 2. Fourteen of the included reviews were systematic reviews and the remaining two^23^, ^24^ were narrative reviews. Five of the systematic reviews performed a meta‐analysis.^12^, ^25^, ^26^, ^27^, ^28^ Seven reviews^23^, ^25^, ^28^, ^29^, ^30^, ^31^, ^32^ provided findings for knee OA and were published between 2005^31^ and 2014,^29^ with the studies included in those reviews published between 1952 and 2011. Four reviews^26^, ^27^, ^33^, ^34^ provided findings for hip OA and were published between 2008^33^ and 2018,^26^ with the studies included in those reviews published between 1985 and 2014. Three reviews^10^, ^12^, ^35^ provided findings for both knee and hip OA and were published between 2006^35^ and 2013,^12^ with included studies published between 1987 and 2011. The final two reviews^24^, ^36^ considered OA across a variety of joints and were published in 2009 and 2015, with included studies published between 1977 and 2008. For these latter two reviews, only data pertaining to lower limb joints were extracted. Only one review^12^ included a study that examined risk factors for *ankle* OA in physically demanding occupations and another review reported on a single article for foot OA.^24^ Studies included in the 16 incorporated reviews employed cross‐sectional, case‐control, cohort, or case series designs. Both reviewers agreed on the methodological quality on all but one paper, which was settled by a third reviewer. Based on AMSTAR 2, one systematic review^28^ was deemed to be of *high* methodological quality, one to be of critically low methodological quality,^10^ and the others^12^, ^25^, ^26^, ^27^, ^29^, ^30^, ^31^, ^32^, ^33^, ^34^, ^35^, ^36^ of *moderate* methodological quality. Two reviews were not rated because they were narrative (not systematic) reviews.

**Table 2:** Eligible publications and key extracted data, in descending order of methodological rating as determined by the AMSTAR 2 instrument^22^

Author, Year [Reference Number]	Review type; N of included studies, years of publication of included studies	Types of studies included	Focus	Key findings	Methodological rating (AMSTAR 2)
*Knee OA*					
Ezzat & Li, 2014^29^	Systematic review of 32 studies (1952‐2011)	Cross sectional, case‐control and cohort	Relationships between occupational physical loading of various types and knee OA	Moderate evidence that combined heavy lifting and kneeling constitute a risk factor for knee OA; limited evidence for heavy lifting, kneeling, stair climbing, or occupational groups being individual occupational risk factors for knee OA. Elevated BMI and previous injury have a role in the development of knee OA. There was a moderate level of evidence for males but limited evidence for females	Moderate
Jensen, 2008^30^	Systematic review of 25 studies (1952‐2005)	Cross sectional, case‐control and cohort	Occupational risk factors for development of knee OA	Moderate evidence for kneeling and heavy lifting as risk factors for knee OA, with the combination of both associated with greater risk. Stair and ladder climbing were also associated with increased risk of knee OA. The evidence was stronger for males than females	Moderate
McMillan & Nichols, 2005^31^	Systematic review of 19 studies (1952‐2000)	Case‐control and cohort	Occupational risk factors for knee OA in miners	Work involving kneeling and or squatting is associated with increased risk of knee OA. Frequent or prolonged kneeling or squatting is associated with double the risk of knee OA observed in the general population. Lifting with squatting/kneeling is associated with further increases in risk	Moderate
McWilliams et al, 2011^28^	Systematic review and meta‐analysis of 66 studies (1955‐2010)	Cross sectional, case‐control and cohort	Occupational risk factors for OA of the knee	Occupational activities incorporating kneeling, lifting, carrying, squatting or other knee bending activities are associated with increased risk of knee OA	High
Palmer, 2012^32^	Systematic review of 43 studies (1968‐2010)	Cross sectional, case‐control and cohort	Occupational risk factors for OA of the knee	Good evidence exists that physical work activities incorporating kneeling, squatting, lifting or climbing increase risk of, and can aggravate, knee OA. High BMI also independently related to knee OA	Moderate
Reid et al, 2010^23^	Narrative review of 7 studies (1988‐2008)	Cross sectional, case‐control and cohort	Occupational risk factors for musculoskeletal disorders of the knee, including knee OA	Kneeling and squatting are primary risk factors for knee OA, with crawling, stair/ladder climbing, lifting/carrying/moving, walking and standing up from a knee/squat/crawl also associated with an increased risk of knee OA	Not applicable—narrative, not systematic, review
Silverwood et al, 2015^25^	Systematic review with meta‐analysis of 46 studies (1991‐2011)	Cohort	Evidence for risk factors for knee OA in older adults	Kneeling and lifting were significantly related to knee OA. Heavy physical workload and knee bending not significantly related to knee OA. Previous knee injury, female sex, overweight, and obesity are also risk factors for knee OA	Moderate
*Hip OA*					
Bergmann et al, 2017^27^	Systematic review and meta‐analysis of 23 studies (1991‐2014)	Case‐control and cohort	Relationships between heavy lifting and carrying and hip OA	An association exists between years of heavy lifting and carrying and risk of developing hip OA. The effects were lower for females, possibly due to females being underrepresented in studies	Moderate
Jensen, 2008^33^	Systematic review of 19 studies (1985‐2004)	Cross sectional, case‐control and cohort	Occupational risk factors for development of hip OA	Moderate to strong evidence for heavy lifting being a risk factor for hip OA in farmers. Limited evidence for climbing stairs or ladders as risk factors for hip OA. The evidence was stronger for males than for females	Moderate
Seidler et al, 2018^26^	Systematic review with meta‐analysis of 23 studies (1991‐2014)	Case‐control	Dose‐response relationship between different types of physical workload and OA of the hip	An increased risk of hip OA is associated with heavy lifting and as heavy lifting increases, risk of OA increases. A linear association was found between manual handling of weights and hip OA in males but not females	Moderate
Sulsky et al, 2012^34^	Systematic review of 30 studies (1984‐2009)	Case‐control and cohort	The relationships between physical workloads and hip OA	Heavy lifting is a risk factor for hip OA and long‐term exposure to standing may also increase the risk of hip OA	Moderate
*OA in various joints*					
Fransen et al, 2011^10^	Systematic review of 22 studies (2007‐2010)	Cross sectional, case‐control and cohort	The role of occupational risk factors in the development of knee and hip OA	Men involved in farming or construction are at increased risk of developing chronic hip and knee pain and OA. The risk of knee and hip OA from regular lifting, kneeling and crawling is increased with concomitant obesity	Critically low
Richmond et al, 2013^12^	Systematic review with meta‐analysis of 43 studies (1977‐2008)	Cross sectional, case‐control, cohort and case series	Occupational risk factors for OA in the lower limb	Occupational activity including heavy lifting, squatting, kneeling and climbing stairs is associated with an increased risk of OA at the knee and hip. No evidence for occupational activity and ankle OA. Other factors including obesity and previous joint injury are also associated with an increased risk of hip and knee OA	Moderate
Vignon et al, 2006^35^	Systematic review of 76 studies (1988‐2004)	Cross sectional and case‐control	The relationship between specific occupational activities and knee and hip OA	There is high level of evidence of a positive relationship between physically demanding occupational activity, including heavy lifting and climbing, and knee and hip OA	Moderate
Aluoch & Wao, 2009^36^	Systematic review of 16 studies (1987‐2008)	Cross sectional and case‐control	Occupational risk factors associated with the development of OA in any joints of the body.	Strong relationship between physical strain experienced while performing physically demanding jobs and the incidence of OA of the knee and hip	Moderate
Yucesoy et al, 2015^24^	Narrative review of 30 studies (1988‐2011)	Cross sectional and case‐control	Occupational risk factors for OA	Heavy physical workload is the most common risk factor for OA in several anatomical locations including the knee and hip. Other risk factors include kneeling, regular stair climbing, crawling, bending, whole‐body vibration and repetitive movements	Not applicable—narrative, not systematic, review

Abbreviations: AMSTAR, A MeaSurement Tool to Assess systematic Reviews; BMI, body mass index; OA, osteoarthritis.

### 3.2 Synthesis

Physically demanding jobs have been strongly associated with an increased risk of OA of the hip and knee; however, many studies investigating these relationships have been limited by non‐response bias, small sample sizes, and retrospective exposure assessments.^36^ The physically demanding jobs most often associated with OA of the hip and knee joints are those which entail frequent knee bending, heavy lifting, stair climbing, and prolonged squatting.^12^, ^24^, ^36^ On this basis, the synthesis of evidence from the included reviews that follows is primarily structured around the affected joints, and the types of tasks that have been associated with development of OA in those joints.

### 3.3 OA of the knee

Seven included reviews^23^, ^25^, ^28^, ^29^, ^30^, ^31^, ^32^ reported on occupational risk factors for the development of knee OA, with a further three^10^, ^12^, ^35^ combining OA of the knee and hip (Table 2). Heavy physical work is one of the most common risk factors for the development of knee OA.^24^ Physical work activities including kneeling, squatting, lifting, and climbing have been associated, by a good level of evidence, with development or aggravation of knee OA.^32^, ^35^ Occupational activities which exert high loads on the knee joints or require unnatural body positions (either at end of range or sustained) and cumulative exposures to these sorts of loads and positions may all contribute to OA development.^35^ One meta‐analysis^28^ involving 51 studies with 526,343 participants concluded that occupational factors (eg, heavy lifting, kneeling/squatting, and climbing) could increase the risk of OA by up to 61%, although there was considerable heterogeneity among studies (I^2^ = 84%) and evidence of publication bias. That review, by McWilliams et al,^28^ was the only included review assessed to be of high methodological quality.

#### 3.3.1 Heavy lifting and OA of the knee

Four reviews^10^, ^29^, ^30^, ^32^ examined the relationship between occupational heavy lifting and the development of knee OA. Jensen's review^30^ in 2008 found a moderate level of evidence for a relationship between heavy lifting and the development of knee OA (odds ratio [OR] heavy lifting/no heavy lifting 1.9‐7.31). A total of 9 of 17 included studies in their review showed a significantly increased risk of knee OA associated with heavy lifting, with a dose‐response relationship evident whereby higher risks were found among those who had greater exposure to heavy lifting (either heavier weight or more frequently) than those with less exposures. In the studies reviewed by Jensen,^30^ reported lift loads were varied, including >10 kg, >25 kg, or >50 kg. Fransen et al^10^ subsequently updated Jensen's 2008 review^30^ in 2011 with the addition of another eight studies, six of which found significant associations between heavy lifting and the development of symptomatic knee OA (OR heavy lifting/no heavy lifting = 1.4‐5.0), though they provided no mention of the frequency or duration of these lifts.^10^ The review of Ezzat 29 in 2014 found only limited evidence for the relationship between heavy lifting and development of knee OA, though the ORs similarly ranged from 1.4 to 7.3 when comparing exposed to non‐exposed personnel in the 32 studies which they reviewed. Ezzat^29^ considered the evidence “limited” because of a lack of cohort studies, bias, confounders, and methodological flaws in the studies they reviewed. Palmer's review^32^ in 2012 determined there was reasonably good evidence indicating occupational lifting caused or aggravated knee OA, with 8 of 14 included studies demonstrating significant risk ratios greater than 1.5 when comparing exposed to non‐exposed personnel. Silverwood et al^25^ found lifting to be significantly related to knee OA in one of the three studies in their review. Cumulative tonnages of lifting which need to occur for knee OA risk to increase were not provided in any of the reviews.

#### 3.3.2 Heavy lifting with kneeling and squatting and OA of the knee

Fransen et al^10^ further explored the interaction of occupational heavy lifting with concurrent kneeling or squatting in relation to the development of knee OA and found exposure to the two factors together (heavy lifting with either kneeling or squatting) increased the risk of developing knee OA, with a mean increase in ORs [95% CI] from 2.4 [1.1‐5.0] for exposure to lifting alone to 3.4 [1.8‐6.3] for exposure to lifting combined with squatting or kneeling. The review by Ezzat 29 also investigated the interaction between heavy lifting and kneeling and found moderate level evidence indicating that exposure to these combined occupational tasks contributed to the development of knee OA, with the associated ORs in their review ranging from 1.8 to 7.9 when comparing exposed to non‐exposed personnel. McMillan et al,^31^ in their 2005 review, found similar interactions between heavy lifting and prolonged knee bending or squatting or repeated knee bending in increasing the risk of developing knee OA, noting that these factors together were associated with a greater risk of developing knee OA than knee bending activities alone. This relationship was further supported by Palmer's later review,^32^ which found that risks of developing knee OA were elevated three‐ to eightfold when lifting was combined with kneeling or squatting.

#### 3.3.3 Kneeling, squatting, and crawling and OA of the knee

Silverwood et al^25^ deemed occupational kneeling to be an important risk factor for OA, but in the review by Ezzat^29^ only limited evidence supported the relationship between occupational kneeling alone and the development of knee OA. Despite 11 of the 16 studies included which examined occupational kneeling in the Ezzat review^29^ showing significant associations between occupational kneeling and development of knee OA (OR exposed/non‐exposed 1.5‐6.9), only nine studies were deemed to be of high methodological quality and six of those showed positive associations of this nature, with the other three not showing significant relationships. Jensen^30^ found that 8 out of 12 studies reviewed identified a significant association between squatting for greater than 1 hour per day and development of OA in the knee. They concluded exposure to squatting led to a two‐ to sevenfold increase in the odds of developing knee OA, based on what they deemed to be a moderate level of evidence.^30^ The subsequent update of Jensen's review^30^ by Fransen et al^10^ supported a significant twofold increase in the risk of people developing painful knee OA when exposed to kneeling or crawling at work. The exposures in the studies reviewed by Fransen et al^10^ were squatting for greater than 30 minutes per day or in total for greater than 15% of the work day.

McMillan et al,^31^ in their review, aimed at determining the occupational risk factors for knee OA in miners, concluded that kneeling and squatting are causally associated with an increased risk of developing OA of the knee. They estimated that occupations which required frequent or prolonged kneeling or squatting doubled the risk of people developing OA of the knee when compared to the risk observed in the general population.^31^ Palmer's review^32^ found 11 of 17 studies reported significant relationships between work activity involving kneeling or squatting and the risk of developing knee OA, with associated relative risks >1.5. It should be noted, however, that only 1 of the 17 studies was a cohort study, with the rest being case‐control or cross‐sectional studies.

#### 3.3.4 Climbing and OA of the knee

Jensen's review^30^ found only limited evidence to support the relationship between climbing stairs at work and the development of knee OA, while the evidence for a relationship between climbing ladders and development of knee OA was deemed to be inconclusive. The associations identified in that review, despite being significant, were all from case‐control studies, with the retrospective nature of this methodology making the studies prone to both recall and selection bias. The authors of the review nevertheless acknowledged that climbing stairs may be an aggravating factor for those who have stairs at work. Upon updating Jensen's earlier review,^30^ Fransen et al^10^ concluded that little evidence remained that climbing stairs or ladders was associated with the development of symptomatic knee OA. In line with those findings, Ezzat^29^ also concluded that only limited evidence existed to suggest stair climbing was a risk factor for knee OA (OR exposed/non‐exposed 1.6‐5.1), with one study included in their review suggesting a protective effect of stair climbing against the development of knee OA.

### 3.4 OA of the hip

Seven included reviews^10^, ^12^, ^26^, ^27^, ^33^, ^34^, ^35^ reported on occupational risk factors for the development of hip OA (Table 2). Similar to the results of the reviews focused on the knee, occupations which entail specific types of physical strain while completing physically demanding tasks have been found to have a strong relationship with the incidence of hip OA.^12^, ^24^, ^35^, ^36^ In a similar manner to the evidence pertaining to the knee, some evidence supports the relationship between occupational activity including heavy lifting and the development of hip OA.^35^ In contrast to the knee, however, hip OA seems predominantly related to forces exerted on the hip joint through heavy lifting as opposed to high loads on the joint from other mechanisms, unnatural body positions, and other types of cumulative exposures that are associated with occupational knee OA.^35^

#### 3.4.1 Heavy lifting and OA of the hip

Occupational lifting has been found to be associated with the development of hip OA. Bergmann et al^27^ found roughly a 150% increase in risk (relative risk = 2.46) of developing hip OA for men who were exposed to heavy occupational lifting, with a dose‐response relationship indicating that greater exposures to lifting were associated with greater levels of risk. In the studies included in their review, loads ranged from 4 kg to more than 40 kg, with a minimum dose of 20 kg lifted regularly required to increase the risk of hip OA over 20 years of exposure.^27^ Risk of developing hip OA was found to be increased after only 10 years of lifting loads of around 50 kg, or 20 years for regular lifts of 20 kg.^27^ The cumulative loading threshold was reported to be 3000‐5000 tonnes to increase the risk of hip OA significantly.^27^ No indication was given as to how many lifts per day were required, however, if using 3000 tonnes lifted, 20 kg at a time, this would equate to 150 000 lifts. Over a 20 year period this would equal 7500 lifts per year or, if using 220 work days per year, 34 lifts per work day.

Seidler et al^26^ used a similar approach whereby an external reference population was used to determine the dose‐response relationship between physical workload and hip OA. They found three types of cumulative exposure which would double the risk of developing hip OA when compared to the risk for those who were not exposed to lifting. These included lifting 10 100 tonnes of weight comprised of loads >20 kg, 9500 tonnes of loads ≥20 kg lifted more than 10 times per day, or 321 400 movements of weights ≥20 kg. Seidler et al's^26^ review findings were summarized as follows: assuming a 40‐year career duration, a doubling of risk of hip OA would result from lifting between 6100 and 14 000 cumulative tons of weights >20 kg, lifting 6000 to 105 000 cumulative tons of weights >20 kg >10 times per day, or between 218 000 and 514 000 cumulative lifting and/or carrying operations of loads of any weight.

In Jensen's review,^33^ which was published in 2008, moderate to strong evidence was found for a positive relationship between occupational heavy lifting and the risk of developing hip OA, where the burden of lifting involved loads of 10‐20 kg lifted repeatedly for at least 10‐20 years.^33^ There were, however, few studies in that review which mentioned the frequency of these lifts. A total of 12 of the 14 studies included in that review showed a significantly increased risk of hip OA was associated with such heavy lifting, with OR (exposed/non‐exposed) ranging from 1.97 to 8.5. In addition, a dose‐response relationship was found, such that those who were considered to have a high exposure to lifting, reported either by interview or questionnaire, had a higher risk of developing hip OA (OR 1.5‐12) than those who reported a medium exposure to lifting (OR 1.1‐4.1). This risk differential was related to the loads lifted, the frequency with which the loads were lifted, and the duration of lifting. For example, those who lifted more weight were at higher risk of developing hip OA, with ORs of 1.2‐1.9 for lifts >10 kg, ORs of 1.5‐2.7 for lifts >25 kg, and OR’s of 3.2‐8.5 for lifts >50 kg, when compared to lifting loads <10 kg.^33^

Fransen et al's^10^ update to Jensen's review^33^ included an additional eight studies, again finding a significant association between heavy lifting and hip OA (OR’s exposed/non‐exposed 1.7‐6.7). The lifting exposures sufficient to increase the risk of hip OA have been reported to be as low as 10 kg or more lifted from one to 10 times per week (no threshold duration reported).^36^ Sulsky et al's review^34^ identified evidence for the relationship between heavy lifting and risk of developing hip OA but failed to identify the dose‐response relationship reported by Seidler et al^26^ and Jensen^33^ within the literature they reviewed. Only 6 of the 30 studies reviewed by Sulsky et al^34^ provided quantitative exposure data, and only three of those six were deemed to be of good methodological quality.

#### 3.4.2 Lifting with squatting or standing and OA of the hip

No significant association was found between hip OA and combined lifting and squatting in the review of Fransen et al^10^ The combination of occupational heavy lifting and standing was explored in the review of Sulsky et al,^34^ which found an increased risk of hip OA was associated with standing and heavy lifting (10‐25 kg) at work over the long term, but this increase in risk was determined to be small and the authors highlighted that there was a high variability in the results reported in included studies.

#### 3.4.3 Climbing and OA of the hip

Jensen's review^33^ also examined the relationship between climbing stairs or ladders and the risk of subsequently developing hip OA. Despite three of five studies demonstrating a significantly increased risk of hip OA with climbing (ORs exposed/non‐exposed 2.3‐2.5), the high quality study in the review did not show a significant association, and therefore, the evidence for a causal relationship was deemed to be limited.^33^ The findings of Fransen et al's^10^ subsequent review mirrored this result, with only one of three studies demonstrating a significant association between climbing and the risk of developing hip OA.^10^ The review of Sulsky et al^34^ similarly reported that long‐term exposure to stair climbing may be associated with hip OA but noted that results were inconsistent across studies.

#### 3.4.4 Crawling, kneeling, squatting and sitting and OA of the hip

Limited evidence was provided by one review for a relationship between occupational crawling and the development of hip OA.^10^ There was no evidence reported by any of the included reviews to explore the potential relationship between sitting, kneeling, or squatting without lifting and the development of hip OA.

### 3.5 OA of the ankle

A single study which reported on associations between OA of the ankle and occupational activity was found in the review by Richmond et al^12^ In that study no association was found between the number of descents performed by military parachutists and development of ankle OA.

### 3.6 OA of the foot

The narrative review by Yucesoy et al^24^ reported on a single article pertaining to occupational risk factors for foot OA. Stair climbing was reported to be associated with foot OA, however, no exposure duration or dosage was provided.

### 3.7 Additional factors

In addition to occupational factors, there are other factors which were shown in the included reviews to contribute to an increased risk of personnel in physically demanding occupations developing lower limb OA. Gender, older age, obesity or high body mass index (BMI), previous injury, and sporting activity have all been linked to the development of knee OA,^23^, ^25^, ^29^, ^31^, ^32^, ^37^ hip OA,^36^ and both knee and hip OA.^12^, ^35^ In two of the included reviews,^30^, ^38^ males appeared to be at greater risk of developing knee OA. Despite Silverwood et al^25^ reporting that females were found to be at a higher risk for knee OA than males, it should be noted that this was in the general population and not due to occupational tasks. Most reviews agree that females are underrepresented in the occupational literature at this point in time, which may explain the apparent elevated risk among males.^27^, ^29^

Older age has also been associated in the included reviews with a sharp increase in incidence of knee OA, particularly between the ages of 50–75 years, and a leveling off above the age of 75–80.^25^ Likewise, overweight or obesity, typically reported as a high BMI (>25), has been associated with an increased risk of OA in numerous included reviews,^10^, ^12^, ^25^, ^29^, ^32^ with ORs of 2.10–2.66 reported when comparisons are made to those with what is considered normal BMI.

Previous injury is a known risk factor for OA,^12^, ^29^ with a pooled OR of 2.83 when previously injured personnel in physically demanding occupations were compared in one of the included reviews to those who have not been previously injured,^25^ though the level of heterogeneity was high (*I* ^2^ = 89%). The association between sporting activity and OA is contentious in these occupational populations, with mixed results in reviews,^12^ various sports studied,^35^ high levels of heterogeneity^12^, ^35^ and at least some of the risk being explained by previous injury within sport.^35^ The OA risk associated with sports participation appears to be far less than the OA risks associated with previous injury and being overweight.^35^ Estimates are that high BMI in conjunction with previous injury may increase the risk of developing knee OA by 5‐ to 15‐fold,^32^ a much greater increase in risk than the two to four times risk increase associated with sporting activity and dependent on the sport.^12^

### 3.8 Limitations of included reviews

There are several possible reasons for the varying results, where these occurred, across the included reviews and these are summarized in Table 3. The issues related to differing diagnostic criteria for OA were by far the most prevalent. Included studies from reviews variably used a radiographic diagnosis, a clinical diagnosis, or a combination of both.^32^, ^35^ Within radiographic diagnoses there have also been differences, with variations in the the K/L scoring (2‐4 or 1‐4) reported to dilute the true incidence of OA.^30^ In addition, the design of the studies included in the various reviews has likely impacted the conclusions. Despite prospective cohort studies being ideal, they are expensive and take considerable time to implement.^23^, ^28^ Numerous retrospective studies are therefore covered in these reviews, with a subsequent loss in methodological quality and hence in the validity of study findings.^23^

**Table 3:** Limitations of studies in reviews

Diagnostic criteria for OA Clinical vs radiographic vs both Kellgren & Lawrence (K/L) scoring variations
Study Design Mostly retrospective Few prospective cohort studies
Sampling Convenience samples often used Potentially biased groups (eg, clinical groups awaiting knee replacement)
4) Lack of control for co‐variates known to affect OA, for example, BMI, previous injury, sporting participation
5) Varying definitions of occupation and exposures 6) Few report a minimum exposure duration 7) No studies on the military

Abbreviations: BMI, body mass index; OA, osteoarthritis.

A further reason for variations in findings of the included reviews may be the sampling bias in reviewed studies. For example, some studies used convenience samples at orthopedic clinics,^36^ which may result in for example, ethnic groups with a lower prevalence of OA^33^ or high proportions of farmers who undertake high physical workloads and not representative of the general population. In addition, using samples of those who are on a wait list for surgery or those who have already undergone joint replacement surgery may also give rise to bias.^34^ Some reviews included studies where authors did not control for the individual's activities of daily living, sport participation, age, BMI, or previous injury, all of which are known to influence results, and the reviews themselves did not conduct sensitivity analyses to explore how findings might have been affected by inclusion of these studies.^23^, ^36^ One review utilized only one reviewer for the study selection, data extraction and quality assessment elements of the review and limited their search to only two databases.^29^ In this same review, “occupation” and “occupational exposure” were poorly defined.^29^ For example, despite a homemaker role possibly requiring heavy lifting, squatting and carrying, it was not recorded as an occupation per se, despite representing a similar exposure to a paid job involving manual labor.^29^

Studies considered in the included reviews demonstrated discrepancies in what they considered to be “heavy” with respect to lifting (10 kg, 25 kg, 50 kg, etc) and in whether lifting frequency was reported per day, per week, or over a lifetime of work. Some studies considered in the included reviews classified exposures to lifting as “low,” “moderate,” or “high,” when determining associations between heavy lifting and risk of developing OA, without adequately defining these levels.^29^ Likewise, quantification of climbing has varied across studies considered in the included reviews, and it has been variably reported in terms of duration (eg, >30 mins/d), absolute numbers of times each day that stairs are climbed (eg, >30 times per day), or numbers of flights of stairs climbed (eg, 15 flights of stairs/d), making direct comparisons difficult.^30^ There are also inherent difficulties in associating exposures to specific occupational activities, which may vary over time, with development of OA, due to latencies in the development of symptoms or radiographic change associated with OA. Other difficulties are evident in studies considered in the included reviews. These relate to poor definition of the retrospective time frames in which exposures have occurred and recall bias that occurs in retrospective accounts of exposed groups, which tend to inflate reported exposures, especially if participants have been tasked with recalling decades of exposures.^34^, ^35^

In addition, the healthy survivor worker effect should be acknowledged, whereby exposure data may be influenced by the early departure from the workforce of those who developed OA early in their career and subsequently left the industry, leaving personnel in the workforce who were less affected by OA, but contributed many more years of exposure in the overall workforce exposure calculations.^39^ Likewise, those who gravitate to physically demanding jobs may be fitter, with less joint disease than those in the more sedentary populations to which they are typically compared.^32^ Conversely, those who are tasked with physically demanding jobs may be affected more by their OA and subsequently seek treatment earlier than those in more sedentary occupations.^32^ These factors may have affected the findings of many of the included reviews.

## 4 DISCUSSION

The included reviews found moderate to good evidence that heavy occupational lifting is associated with an increased risk of OA at the knee^10^, ^28^, ^29^, ^31^, ^32^, ^36^ and the hip.^10^, ^26^, ^27^, ^33^, ^34^, ^36^ The definition of “heavy” has ranged from 10 kg^36^ up to 50 kg.^33^ Despite no cumulative lifting threshold being found for the knee, cumulative tonnes of lifting associated with significantly increased risks of hip OA have been reported to be between 3000^27^ and 14 000^26^ tonnes of weight for lifts of 20 kg. In addition, the combination of heavy lifting and physically demanding occupational tasks such as kneeling^30^ or squatting^40^ appears to further increase the risk of developing knee OA.

The results of numerous included reviews^23^, ^28^, ^31^, ^32^, ^35^, ^36^ suggest that squatting appears to be associated with knee OA when excessive exposures exist in occupations. The concerns with squatting are for estimated peak external moments created at the knee during squatting, which are up to 2.5 times greater than those experienced when walking.^41^ These forces can have long‐term implications for both mechanical functions of the knee joint and for structural integrity of cartilage within the joint.^42^

In a similar manner, activities which require knee bending or kneeling have been well investigated and overall appear likely to be related to the development of knee OA.^10^, ^23^, ^24^, ^28^, ^29^, ^30^, ^31^, ^32^, ^35^, ^36^ Kneeling concentrates around 70% of body weight on a small surface of the tibia and patella, which may damage articular cartilage.^30^ Workplace interventions have therefore been suggested to minimize the frequency and duration of knee bending activities; however, the difficulties associated with implementation of such changes have been acknowledged.^32^

Climbing has been identified as a factor that contributes to knee OA.^24^, ^29^, ^32^, ^35^, ^36^ However, the evidence suggesting that climbing ladders or stairs influences development of knee OA is limited.^29^ Climbing has also been implicated in the development of hip OA in several reviews.^10^, ^33^, ^35^, ^36^ Forces of up to six times body weight are experienced during stair climbing,^30^ with an element of rotational loading.^43^ Difficulty remains in quantifying the threshold, if any, beyond which climbing may contribute to development of knee or hip OA, as some studies have found it to have a protective effect.^44^, ^45^

There are some military specific tasks that may give rise to knee pain and/or injuries, which then have potential to lead to longer‐term issues such as OA. In soldiers undertaking prolonged mounted patrolling in Afghanistan, up to 33% of soldiers reported knee pain, with significant associations between this pain and the amount of time they spent on vehicles each week.^46^ If this pain reflects underlying joint injury, then the findings of this umbrella review indicating that prior injury is a risk factor for development of lower limb OA would suggest that exposure to such tasks may increase the longer‐term risk of military personnel developing lower limb OA. A potential source of knee symptoms and contributor to knee OA among naval personnel is the steep inclination angles of naval ladders, which have been shown to increase knee flexion angles and expose the knee to joint forces equating to up to 6.6 times body weight.^47^

Given that military occupations typically require carrying heavy loads, heavy lifting, walking, crawling, kneeling, and squatting, often for extended periods of time under conditions of caloric and sleep deficit, it is not surprising that there are relatively high rates of OA among military personnel.^14^, ^18^, ^46^ Control of risk is a difficult concept in this context, as military training must mimic occupational demands, with chronic physical and mental conditioning vital for achieving mission tasks. Given that load carriage, crawling, kneeling, and squatting are essential requirements in the military domain, avoiding these activities is not possible or desirable since training must closely replicate expected combat/occupational actions. Primary prevention could more reasonably be focused on attempting to decrease loads where appropriate,^48^ minimizing initial injuries where possible^49^ by ensuring adequate strength around affected joints^50^ and maximizing fitness^51^ and ensuring complete rehabilitation of injuries when they do occur. Additional risk factors could include gender (females),^52^ age (older),^25^ years of service (longer),^18^ BMI (high),^29^ aerobic fitness (low), and strength (low), all of which may negatively affect the relationship between occupational risk factors and the risk of developing lower limb OA or experiencing injuries that may predispose personnel to lower limb OA.

Military specific risk factors for development of OA appear to include ground‐based service and higher rank.^11^, ^20^ Given the length of service required to reach higher ranks and the greater exposure to physical demanding tasks that might be expected during this time, length of service may also be associated with an increased risk of OA as a proxy measure of length of service.^18^ Ground‐based service often involves navigating difficult terrain while wearing heavy fighting loads and being physically engaged in conflict, or simulated conflict during training. These features of ground‐based service may help explain and contribute to the increased risk of developing OA associated with such service.

Quantifying what constitutes a protective rather than detrimental exposure is a vital step in minimizing the impacts of lower limb OA in physically demanding occupations. Further scrutiny of specific thresholds of weights lifted and carried, and cumulative durations spent crawling, squatting or kneeling over the time period in specific occupations is required based on the finding of this review.

## 5 CONCLUSIONS

The results of this review suggest that occupations which involve heavy physical workloads increase the risk of developing lower limb OA. Heavy lifting, squatting, knee bending, kneeling, and climbing may all increase the risk of developing OA in both knees and hips. Where possible, efforts should be made to decrease the quantity and durations of these tasks, and to pursue preventative measures such as muscle strengthening, ensuring optimal BMI, injury minimization, and complete rehabilitation of previous injuries.

## DISCLOSURE

*Approval of the research protocol*: N/A. *Informed Consent*: N/A. *Registry and the registration number of the study/trial*: N/A. *Animal studies*: N/A. *Conflict of interest*: The authors declare no conflict of interests for this article.

## AUTHOR CONTRIBUTIONS

BS, RO, RP, EC, and JK were involved in conception, design, acquisition, analysis, and interpretation of the data. All authors were involved in drafting the work and revising it critically and gave final approval. BS was involved in accountability for the work.
